# Role of Intermittent Fasting in the Management of Prediabetes and Type 2 Diabetes Mellitus

**DOI:** 10.7759/cureus.28800

**Published:** 2022-09-05

**Authors:** Tioluwani K Ojo, Olajide O Joshua, Oboseh J Ogedegbe, Oluwapelumi Oluwole, Ayoade Ademidun, Damilola Jesuyajolu

**Affiliations:** 1 Internal Medicine, Benjamin S. Carson (Snr) College of Health and Medical Sciences, Ilishan-Remo, NGA; 2 Internal Medicine, Lifeway Medical Centre, Abuja, NGA; 3 Medicine and Surgery, Benjamin S. Carson (Snr) College of Health and Medical Sciences, Ilishan-Remo, NGA; 4 Surgery, Olabisi Onabanjo University Teaching Hospital, Sagamu, NGA; 5 Neurosurgery, Association of Future African Neurosurgeons, Yaoundé, CMR

**Keywords:** glycated hemoglobin (hba1c), fasting blood sugar, religious fasting, therapeutic fasting, prevention of diabetes, intermittent calorie restriction, types 2 diabetes, pre-diabetes

## Abstract

Diabetes mellitus (DM) is a highly prevalent disease in the modern society. It can be defined as a group of metabolic diseases marked by chronic hyperglycemia arising from defects in insulin secretion or resistance to insulin action, or both. Its predecessor, prediabetes, is also an important entity, and its management is essential to prevent its progression to DM. Together, these entities burden global health and the world economy, and therefore, prevention and management are key to improving global health and reducing the financial burden on the world economy. Comprehensive lifestyle modification has been proven to be a safe and effective method for preventing the progression of prediabetes and treatment of type 2 diabetes mellitus. Lifestyle modifications such as weight loss, exercise, and diets such as low-carbohydrate one, Mediterranean, and very low calorie diets are traditionally recommended. These particular diets aim to attain calorie deficits and thus induce weight loss. Intermittent fasting (IF) is one such diet that focuses more on the timing of calorie consumption. However, there are several methods of achieving this, which are highlighted in this review. IF has been shown to promote weight loss, reduce insulin resistance, improve glycemic control and lower the risk of cardiometabolic diseases. However, little literature is available regarding the use of IF in managing DM. This review intends to elucidate the role of intermittent fasting in preventing and treating DM, including its benefits and limitations. From the various studies reviewed in this article, it can be deduced that intermittent fasting can achieve suitable glycemic targets and weight control.

## Introduction and background

Diabetes mellitus (DM) is a group of metabolic diseases marked by chronic hyperglycemia arising from defects in insulin secretion or resistance to insulin action, or both, with associated abnormalities in carbohydrate, protein, and lipid metabolism. The World Health Organization (WHO) also estimates that about 422 million people worldwide have DM, and the majority are living in low- and middle-income countries. DM is linked directly to 1.5 million deaths yearly [1]. The American Diabetes Association (ADA) has stated that a fasting plasma glucose level of ≥126 mg/dL (7.0 mmol/L), plasma glucose level of ≥200 mg/dL (11.1 mmol/L) two hours following the oral glucose tolerance test (OGTT), hemoglobin A1C (HbA1c) ≥6.5%, or a random plasma glucose level of ≥200 mg/dL (11.1 mmol/L) together with symptoms of hyperglycemia are all diagnostic of DM [2]. There are three established classifications of diabetes mellitus used in clinical practice: type 1 diabetes mellitus (T1DM), type 2 diabetes mellitus (T2DM), and gestational diabetes mellitus (GDM). T1DM arises when there is a deficient secretion of insulin from the pancreas, usually secondary to an autoimmune process, while T2DM and GDM arise due to a resistance to insulin action [1,2]. Prediabetes (intermediate hyperglycemia) is a high-risk state for T2DM, defined by glycemic parameters that are greater than normal but lower than established DM thresholds. Studies have shown that 1 in 10 people with prediabetes progress to DM every year, with the same proportion converting back to normoglycemia [3]. Patients can be said to have prediabetes if they fulfill any of the following criteria: impaired fasting plasma glycemia (IFG) values ranging from 100 to 125 mg/dL (5.6-6.9 mmol/L) or an impaired glucose tolerance test with two-hour plasma glucose values in the oral OGTT ranging from 140 to 199 mg/dL (7.8-11.0 mmol/L). An HbA1c value of 5.7%-6.4% can also be used to diagnose prediabetes, as recommended by the ADA [2].

DM also poses a tremendous financial burden on the world's economy. In 2017, the estimated cost of diagnosed DM in the United States was 327 billion dollars [4]. Therefore, there is a need for more cost-effective approaches to DM management. Weight loss has been proven to be an effective method to prevent and improve the management of T2DM for individuals who are overweight or obese. Along with other lifestyle factors like exercise and behavior modification, diet plays a central role in weight loss. Several popular diets positively affect weight loss, DM, and cardiovascular risk factors. This review attempts to elucidate the role of one such dietary practice, termed intermittent fasting (IF), in preventing and treating T2DM. Regardless of the macronutrient content, diets with equivalent caloric intakes result in similar weight loss and glucose control [5]. IF focuses more on the timing of caloric intake, but a calorie deficit is usually accompanied by undergoing intermittent fasting. Individuals typically undergo intermittent fasting for personal or religious reasons. The Ramadan observed by Muslims is an example of a religious basis for intermittent fasting [6].

## Review

Physiology of fasting

Glucose is the body's primary source of fuel in non-fasting states. Within the initial 12-24 hours of fasting, the human body attempts to maintain blood glucose levels by breaking down glycogen stores in the liver and skeletal muscle. This is stimulated by glucagon, an endogenous hormone secreted from the alpha cells of the pancreatic islets of Langerhans. The process of converting glycogen to glucose is termed glycogenolysis. Beyond 12-24 hours, the body's glycogen storage is often depleted. It then resorts to breaking down fatty acids (lipolysis) to produce energy. Triglycerides are broken down into glycerol and fatty acids by hormone-sensitive lipase, an enzyme whose activity is increased by glucagon, epinephrine, and cortisol. Fatty acids are then transported to the liver, where they undergo beta-oxidation in the mitochondria to become acetyl CoA. Acetyl CoA is then converted to acetoacetate and beta-hydroxybutyrate (ketone bodies) through a process termed ketogenesis. Ketogenesis is strongly suppressed by insulin. This is important because insulin is deficient in type 1 diabetics and, to a lesser extent, in T2DM. Insulin deficiency can lead to excessive ketone body production, along with elevated levels of plasma glucose; these constitute key features of diabetic ketoacidosis. In the absence of insulin or the presence of insulin resistance, gluconeogenesis in the fasting state is left unchecked, combined with an inability to transport glucose into cells, resulting in an elevated plasma glucose level, which can be used to diagnose impaired fasting glucose (prediabetes) or DM [7,8].

What is intermittent fasting?

Intermittent fasting, also called intermittent energy restriction (IER), is a widespread dietary practice consisting of alternating periods of liberal dietary consumption and abstinence from caloric intake. Studies have shown that IF has short- and long-term benefits for health that go beyond enhancing weight loss [9]. In other words, IF involves a complete or partial restriction of energy within defined brief time frames on a recurrent basis. Its principle consists of taking periodic eating breaks to induce energy deficit and improve metabolism. The American Heart Association states intermittent fasting may produce weight loss, lower the risk of cardiometabolic diseases, and reduce insulin resistance, although its long-term reliability is unknown [10]. There are many ways IF can be achieved to reduce total calorie intake; these are listed in Table 1 [11,12].

**Table 1:** Common intermittent fasting regimens

Intermittent fasting regimen	Description
Periodic fasting	Fasting for up to 24 hours once or twice a week with ad libitum (as often as necessary or desired) food intake for the remaining days.
Time-restricted feeding	Involves eating for only 8 hours and fasting for the other 16 hours of the day.
Alternate-day fasting	Involves fasting for 24 hours with subsequent ad libitum feeding for the next 24 hours. Fasting days are typically achieved by consuming 0-25% of daily caloric needs.
The 5:2 diet	A derivative of the periodic fasting method, it involves restricting caloric intake on two non-consecutive days a week (500 kcal for women and 600 kcal for men), with sensible eating, but no formal energy restrictions on the remaining days.

It has been said that intermittent fasting has an impact on metabolic regulation due to effects on modifiable lifestyle behaviors, circadian biology, and the gut microbiome [13]. In humans, studies have shown that insulin sensitivity varies according to the time of the day, with values decreasing towards nighttime. Mechanisms behind this diurnal variation in insulin sensitivity are still uncertain, but changes in free fatty acid metabolism and availability have been implicated [14]. Excluding energy intake in the evening and nighttime synchronizes food intake with an effective postprandial hormonal release, leading to improved circadian clock gene expression, reprogramming molecular mechanisms of energy metabolism, and improved body weight regulation [13]. This subsequent reduction in energy intake is usually about 10%-30% from baseline, leading to a 1%-8% decrease in weight loss [15]. A study on the effect of alternate-day fasting (ADF) and periodic fasting (PF) on preventing cardiovascular diseases (CVDs) postulated a weight loss reduction of 3%-8% over 3-24 weeks. In addition, a significant reduction in serum cardiovascular markers, which includes a reduction of up to 21% in total cholesterol, a reduction of up to 32% in low-density lipoprotein (LDL) cholesterol, and a reduction of up to 42% in triglycerides, was also noted [16].

Several studies have shown that when people with T2DM practice ADF for at least 11 months, there would be an average reduction of HbA1c of at least 3% [17]. The microbiome, which exists in all living organisms, plays a vital role in homeostasis, metabolism, and nutrition. Recent evidence indicates that IF may lead to remodeling and increased taxonomic diversity in the human gut microbiome [17]. The effects of intermittent fasting on blood sugar have been emphasized in several studies that have demonstrated favorable effects of IF on weight reduction and mitigating metabolic risk factors. This is due to the metabolic shift during the fasting state from glucose utilization to fatty acids and ketone (beta-hydroxybutyrate, acetoacetate, and acetone) metabolization as the body's preferred fuel source. The body deploys ketone bodies and free fatty acids as its primary energy source (lipolysis), slowing down lipid synthesis and storage (lipogenesis) [18].

Current guidelines for the management of prediabetes and T2DM

The preferred first-line management approach for prediabetes is intensive lifestyle modification. Lifestyle changes would include reducing and modifying caloric intake to attain weight loss in persons who are overweight or obese, avoiding tobacco products, doing judiciously prescribed physical activity, limiting alcohol consumption, having adequate quantity and quality of sleep, and stress reduction [19]. Meanwhile, first-line therapy for managing T2DM will depend on the comorbidities of the patient, patient-centered treatment factors, and management needs. However, it generally involves metformin and comprehensive lifestyle modification. Weight loss is also an important management tool in managing T2DM, given its safety and the strength of evidence for its efficacy in improving glycemia and reducing the risk of CVDs. Traditional recommendations have been to use stepwise addition of medications to metformin to maintain HbA1c targets. This approach is advantageous as it provides a clear assessment of new drugs' positive and negative effects and reduces potential side effects and expenses. Other medications (sodium-glucose cotransporter-2 inhibitors, glucagon-like peptide 1 receptor agonists, or GLP-1 RAs), with or without metformin, are appropriate initial therapy for individuals with T2DM or individuals at high risk of atherosclerotic CVD, heart failure, or chronic kidney disease. The early introduction of insulin is recommended if the patient exhibits symptoms of ongoing catabolism (weight loss), if features of hyperglycemia are present, or when HbA1c levels are greater than 10% or blood glucose levels as high as 300 mg/dL (16.7 mmol/L) or greater. A patient-centered approach should guide the choice of pharmacologic agents. A physician should consider the effects on cardiovascular and renal comorbidities, efficacy, hypoglycemia risk, impact on weight, cost and access, the risk for side effects, and patient preferences when managing diabetic agents [20]. Popular pharmacological options in treating DM and their individual risk of hypoglycemic episodes are depicted in Table 2 [20].

**Table 2:** Common antidiabetic agents used in managing diabetes mellitus GLP-1, glucagon-like peptide 1; SGLT2, sodium-glucose cotransporter-2; SUR1, sulfonylurea receptor 1; DPP-4, dipeptidyl peptidase-4

Class of medication	Examples	Mechanism of action	Risk of hypoglycemia
Biguanides	Metformin	Activates adenosine monophosphate-activated protein kinase in the liver, causing hepatic glucose uptake and inhibiting gluconeogenesis through complex effects on the mitochondrial enzymes.	Low
Sulfonylureas	Glimepiride, glipizide, glyburide	Sulfonylureas lower blood glucose levels by increasing insulin secretion in the pancreas by binding to SUR1 receptors, which leads to the blockage of ATP-sensitive potassium (KATP) channels.	High
Thiazolidinediones	Pioglitazone, rosiglitazone	Mechanisms of actions include diminution of free fatty acid accumulation, reduction in inflammatory cytokines, rising adiponectin levels, and preservation of β-cell integrity and function, all leading to improvement of insulin resistance and β-cell exhaustion.	Low
Alpha-glucosidase inhibitors	Acarbose, miglitol	Reduce intestinal glucose absorption	Low
GLP-1 receptor agonists	Exenatide, dulaglutide, semaglutide, liraglutide, lixisenatide	Activating GLP-1 receptors in the pancreas leads to enhanced insulin release and reduced glucagon release responses.	Low
DPP-4 inhibitors	Alogliptin, saxagliptin, linagliptin, repaglinide	Inhibit GLP-1 degradation → promote glucose-dependent insulin secretion.	Low
SGLT2 inhibitors	Ertuglifozin, dapagliflozin, canagliflozin, empagliflozin	GLT2 inhibitors provide insulin-independent glucose lowering by blocking glucose reabsorption in the proximal renal tubule by inhibiting SGLT2	Low
Meglitinides	Nateglinide, repaglinide	They bind to the SUR1 receptor on the β-cell, although with lower affinity than sulfonylureas, and stimulate insulin release similarly.	High
Insulin	Lispro, aspart human insulin, NPH/regular, glargine	Bind to insulin receptors and produce similar effects to endogenous insulin.	High

Goals of treatment of prediabetes and T2DM

Controlling prediabetes can significantly slow or halt its progression to DM and prevent complications associated with prediabetes and DM, such as myocardial infarction and stroke. Therefore, screening for prediabetes should be emphasized in individuals with risk factors for DM such as a family history of DM, body mass index (BMI) above normal, previously impaired fasting glucose and glucose tolerance, history of gestational DM, delivery of a macrosomic baby, polycystic ovarian syndrome, and an abnormal lipid profile [21].

The goals of treatment for prediabetes are (a) to prevent progression to DM and associated microvascular complications by achieving glycemic control and (b) to reduce the incidence of cardiovascular events [22]. About a quarter of patients with impaired fasting or glucose tolerance tests progress to T2DM within three to five years. The ADA recommends that people with prediabetes lose at least 7%-10% of their body weight to prevent the progression of prediabetes to T2DM [2,23]. Due to the complex multisystem pathophysiology of T2DM, patient management should involve an individualized and multidisciplinary approach. The treatment goals are to prevent or delay short- and long-term complications and improve the patient's quality of life [24]. Achieving control of the HbA1c to 7.0% or less is preferable as stringent tight values have been shown to be counterproductive towards preventing macrovascular complications. Fasting plasma glucose levels should be 70-130 mg/dL, and oral glucose tolerance values should be less than 180 mg/dL after two hours [25].

Treatment goals in T2DM include preventing acute events such as hypoglycemic and hyperglycemic episodes. Hypoglycemia is an emergency that often arises as a direct result of treatment; hypoglycemia can lead to seizures and death in the short term. In the long term, hypoglycemia can lead to neurologic sequelae such as hemiparesis and dementia [26]. Hyperglycemia in patients with T2DM can lead to a hyperglycemic hyperosmolar state and rarely diabetic ketoacidosis, which are both serious complications that can result in significant morbidity and mortality. Long-term treatment goals in T2DM are to prevent macrovascular complications (cerebrovascular and peripheral diseases) and microvascular complications (retinopathy, neuropathy, and nephropathy) and, ultimately, death [26].

How intermittent fasting can help to achieve the goals of treatment?

IF causes a decrease in the levels of insulin due to lower glycemic levels and an increase in insulin sensitivity. This decreased insulin level causes a reduction in adipose growth, and this helps in weight control in an individual [12]. There is solid and consistent evidence that obesity management can delay the progression from prediabetes to T2DM and is highly beneficial in treating T2DM. In patients with T2DM and overweight or obesity, modest weight loss improves glycemic control and insulin sensitivity and delays the need for glucose-lowering drugs. Dietary energy restriction can substantially reduce HbA1c and fasting glucose and promote sustained DM remission for at least two years [27]. In addition, the rise in insulin sensitivity is associated with a decrease in the development of atherosclerosis. This is because insulin resistance increases C-reactive protein, reduces LDL particle size, and possesses the inherent atherogenic ability, all leading to the development of atherosclerosis and its complications [21]. Therefore, intermittent fasting is a potentially a valuable treatment option because it can reduce the occurrence of adverse cardiovascular outcomes by ameliorating metabolic and inflammatory pathways [8].

A cross-over study was done by Sutton et al. in which eight men with elevated BMI, HbA1c and impaired fasting glucose and glucose tolerance were randomized into two groups. The mean BMI was 32.2 kg/m^2^, IFG level was 102 mg/dL, and OGTT after two hours was 154 mg/dL. The first group was that with early time-restricted feeding in which participants had a feeding period of only six hours, ending at 3 pm, and the control group had a 12-hour feeding period. After five weeks, they were crossed over to the other group. Participants in the early time-restriction feeding group had a low insulin level, increased sensitivity to insulin and β-cell responsiveness, reduced blood pressure, and decreased oxidative stress [28].

Parvaresh et al. did a randomized study on 70 patients with metabolic syndrome having a mean BMI of 31.5 kg/m^2^, placing them in either a modified ADF or a 25% caloric restriction group for eight weeks. Patients in the ADF group were asked to consume a very low calorie diet (75% energy restriction) during the three fast days (Saturday, Monday, and Wednesday). They then had a diet that provided 100% of their energy needs on each feed day (Sunday, Tuesday, and Thursday). The study results showed a more significant reduction in fasting plasma glucose levels, body weight, waist circumference, and systolic blood pressure in the ADF group than in the mere calorie restriction group [29].

An observational study by Arnason et al. found a decrease in body weight and fasting and postprandial glucose levels after two weeks of daily fasting for 18-20 hours in 10 patients with T2DM treated only with metformin [30].

The studies mentioned above suggest that intermittent fasting contributes to achieving glycemic targets and weight control with or without medication. IF significantly affects achieving treatment goals in prediabetes and T2DM than mere calorie restriction diets.

Limitations of IF in the management of T2DM

Studies on the safety and benefits of intermittent fasting with DM are minimal, and so are the medical guidelines on how to manage therapeutic IF in patients with DM [8]. The type of medication an individual is taking for managing one's DM influences the potential risks that intermittent fasting may lead to and, therefore, requires careful attention when formulating the treatment plan. Antidiabetic drugs such as metformin, acarbose, thiazolidinediones (TZDs), GLP-1 RAs, and dipeptidyl peptidase-4 (DPP-4) inhibitors work in a glucose-dependent manner and generally have a low risk of hypoglycemia. Therefore, these drugs usually may not require dose modifications for persons participating in intermittent fasting [6]. Little is known about the possible risk associated with IF in people with DM and other comorbidities such as chronic kidney disease, chronic liver disease, and heart failure. However, it is known that T2DM patients on insulin or sulfonylureas are at an increased risk of hypoglycemia. An increase in the frequency of self-monitoring glucose testing or the adoption of continuous glucose-monitoring devices will be required to improve the safety of IF in people with DM on blood glucose-lowering agents. Also, as can occur with non-diabetic patients, IF may present an increased chance of vitamin and mineral deficiency and protein-energy malnutrition mainly because current IF regimens do not focus on the content of caloric intake but rather the timing of the intake [8]. IF can also predispose one to develop menstrual irregularities, worsening of peptic ulcers, gout, postural hypotension, and cardiac arrhythmias from electrolyte disturbances.

More studies need to be done on the use of IF in managing prediabetes and DM. A healthcare provider should appropriately monitor patients using IF, and treatment should be adjusted based on the patient's peculiarities. Because T2DM is progressive in many patients, the maintenance of glycemic targets with monotherapy is often possible for only a few years, after which combination therapy is necessary [20].

## Conclusions

Studies have shown intermittent energy restriction to be efficacious in preventing and managing prediabetes and DM, with remarkable improvements in the metabolic and cardiovascular biomarkers of individuals with DM. There is a lot more evidence needed in order to incorporate intermittent fasting into established guidelines for the management of DM. However, early evidence suggests that IF can be prescribed or incorporated into the management plans of diabetic patients with considerable benefits. Most diabetic patients who choose to undergo IF for whatever reason can be educated on its positives and limitations in achieving their goals and managing glycemic control. Extensive research is, however, needed on clinical outcomes in children, the elderly, pregnant women, and people with DM with a normal BMI.
